# Biodegradable Films and Edible Coatings Based on Whey Protein Isolate for Extending the Shelf Life of Commercial Strawberries

**DOI:** 10.3390/foods14223980

**Published:** 2025-11-20

**Authors:** Michelle Fernandes da Silveira, Carla Vieira, Raúl Comettant-Rabanal, Sheyla Loayza-Salazar, Leonardo Fernandes, Sheyla Gonçalves, Carlos W. P. Carvalho, Carlos Conte-Júnior, Otniel Freitas-Silva, Lourdes Cabral

**Affiliations:** 1 Postgraduate Program in Food Science, Universidade Federal do Rio de Janeiro, Av. Athos da Silveira, 149, Rio de Janeiro P.O. Box 21941-909, RJ, Brazil; michellefsilveira@hotmail.com (M.F.d.S.); carlavieira@edu.unirio.br (C.V.); 2 Tecnología e Ingeniería de Alimentos y Procesos (CTIAP—UPSJB Filial Ica), Grupo de Investigación en Ciencia, Escuela Profesional de Ingeniería Agroindustrial, Facultad de Ingenierías, Universidad Privada San Juan Bautista, Carretera Panamericana Sur Ex km 300, La Angostura–Subtanjalla, Ica 11004, Peru; raul.comettant@upsjb.edu.pe (R.C.-R.); sheyla.loayza@upsjb.edu.pe (S.L.-S.); lourdes.cabral@embrapa.br (L.C.); 3 Instituto Federal do Rio de Janeiro, R. Sen. Furtado, 121/125, Maracanã, Rio de Janeiro P.O Box 20270-021, RJ, Brazil; leomf84@gmail.com; 4 National Research Center for Agroindustrial Food Technology, Universidade Federal Rural do Rio de Janeiro, Km 07, Zona Rural, BR-465, Seropédica P.O. Box 23890-000, RJ, Brazil; sheylapa1@hotmail.com (S.G.); carlos.piller@embrapa.br (C.W.P.C.); 5 Embrapa Food Technology, Av. das Américas, 29501, Rio de Janeiro P.O. Box 23020-470, RJ, Brazil; otniel.freitas@embrapa.br

**Keywords:** edible protein films, protein food coating, mechanical properties, rheology, physical properties, shelf life, decay, visual quality

## Abstract

This study aimed to create a unique WPI film formulation that would help maintain strawberry quality. Therefore, an edible coating from WPI was developed, and its physical, mechanical, and rheological characteristics were analysed. WPI is a biopolymer residue with attractive barrier characteristics, biodegradability, and neutral taste that can be used as an edible coating on fragile fruits such as strawberries. Key innovations from this research include a comprehensive evaluation of whey as the sole polymeric component in edible coatings for strawberries, assessing its standalone protective potential; improvement of film formulation based on whey proportion; and an inferred shelf-life extension of whey-coated strawberries aligned with commercial acceptability standards, bridging the gap between research and practical application. This study showed that increasing protein proportion reduced the film’s solubility from 47.6% to 22.4%, thus enhancing its water resistance by up to 2-fold. Still, the film became tensile stiffer and more elastic modulus at 50% RH than at 70% RH. The filmogenic solution’s viscosity enhanced from 2.25 at 25 °C to 4.19 Pa.s^n^ at 4 °C, indicating homogeneous coating of the fruit surface at room temperature and its adhesion at storage temperature. During cold storage, WPI coating reduced the mass loss of strawberries from a range of 5.83–16.71% in the control to a range of 2.56–13.22%, thus decreasing the mass loss by up to 2-fold compared to uncoated fruit from the control treatment, which resulted in better visual quality and a 33% extension of the shelf life of commercial strawberries. Overall, WPI films and coatings have the potential to offer a sustainable and effective protective layer for highly perishable and delicate fruits, extending shelf life and, consequently, reducing waste. Together, these properties can revolutionise the fresh produce industry to enhance global supply chain efficiency.

## 1. Introduction

Strawberries (*Fragaria x ananassa* Duch.) are highly perishable fruit due to high respiration and susceptibility to fungal decay, resulting in short postharvest life [1]. Moreover, this fruit is characterised by having an exposed pulp. It lacks a natural protective cover, making it a fragile and delicate fruit that is susceptible to mechanical damage, which compromises its quality during marketing [2]. For this reason, several techniques have been applied to improve the shelf life of strawberries, including films and coatings made from edible polymers, which have been shown to protect this fruit from deterioration [3].

The production of biodegradable films and edible coatings involves three main components: a high molecular weight polymer, such as a polysaccharide, lipid, or protein, to provide structural support; a plasticiser, such as a low molecular weight polyol to reduce film brittleness and a solvent with a pH adjuster can also be used when necessary [4]. Whey protein isolate (WPI) constitutes 20% of total milk protein and is a by-product of the dairy industry [5]. Due to its nutritional attributes, biodegradability, biocompatibility, safety, thermal stability, emulsification, gelling, foaming, and water retention capacity, WPI emerges as a promising alternative to produce edible coatings [6]. Still, WPI contains antioxidant amino acids that can help preserve the colour of fruit during storage.

However, previous studies have reported the testing of whey proteins in combination systems—for example, in composite coatings formulated with chitosan, carboxymethylcellulose, and other polymers, or in single coatings containing functional additives such as essential oils [4,7,8,9,10,11]. It is essential to note that the combination of polymeric and functional materials can significantly impact the behavior of films and coatings [4,12]. Therefore, evaluating the role of each component individually is crucial for achieving a deeper understanding of their effects and supporting further improvements in the development and application of these systems. Indeed, various studies by different authors have demonstrated that simple WPI-based coatings can be more effective in preserving fresh fruits during storage than when combined with other components [7,13,14], highlighting the complex performance of these systems and their dependence on the fruit matrix and storage conditions. Therefore, one of the distinctive contributions of our study is the comprehensive evaluation of WPI as the only polymeric component applied to whole commercial strawberries.

Additionally, the application context presented here differs from that reported in the existing literature. Whey protein-based formulations previously reported have been applied to dehydrated strawberries or to pre-freezing procedures [15,16,17], rather than to fresh strawberries, as in the present study. In this context, the functionality required from coatings and films to preserve quality differs substantially. Indeed, it is well established in the literature that choosing the appropriate coating depends on the type of fruit and the intended preservation purpose [12].

In this context, considering the matrix evaluated in the present study—fresh strawberries—previous studies have reported coatings formulated with whey protein concentrate (WPC) [8,9,10] or delactosed whey permeate [18]. Nevertheless, the higher purity of WPI compared to WPC results in a greater ability to form cohesive and stable networks, improved gas barrier properties (O_2_ and CO_2_), and higher mechanical resistance [19]. These superior properties of WPI suggest a potentially better performance in systems sensitive to moisture and oxidation, such as fresh strawberries, which exhibit high water activity and intense respiratory metabolism. Thus, the scarcity of studies evaluating WPI-based films and coatings for the preservation of fresh strawberries reinforces the relevance of the present work.

On the other hand, achieving optimal film-forming properties with WPI can be complex, requiring specific formulations to improve mechanical strength and adhesion [20]. In line with this, improving film formulation based on the WPI (polymer) to glycerol (plasticizer) ratio for application in fresh strawberries contributes to reducing this gap in the literature.

Taking into account consumer market preferences, they are fundamental in assessing the shelf life of strawberries, as they directly influence the acceptance and commercial viability of the product; the time that strawberries remain visually attractive, with adequate texture and preserved flavour, is a key factor for retailers and consumers, who prioritise fresh, high-quality fruits [21,22]. Additionally, trade standards establish specific criteria for colour, firmness, and lack of defects, determining how long strawberries can be marketed before being discarded [23]. Thus, an effective edible coating must not only extend the shelf life of the fruit but also ensure that its appearance remains within consumer expectations throughout storage (commercial viability), highlighting the importance of preserving it throughout the supply chain [22]. In this scenario, we evaluate the product’s overall acceptability by considering the maximum acceptable threshold for sale, being a key innovation in the inference of shelf-life extension for whey-coated strawberries [24,25,26], bridging the gap between research and real application.

Within this framework, the primary hypothesis of the present work is that the WPI-based edible coating formulated herein can extend the shelf life of commercial strawberries. Thus, the objectives of the present study were to develop biodegradable WPI-based films, to characterize them physically, mechanically, thermogravimetrically, and rheologically, and to evaluate the visual quality of strawberries when an edible coating was applied during refrigerated storage.

## 2. Materials and Methods

### 2.1. Plant Material

Fresh strawberries (*Fragaria x ananassa* Duch.) were purchased in 250 g rigid plastic containers, each containing 15 to 17 berries with an average weight of 18 g, for a total of 3750 g, from a local supermarket in Rio de Janeiro, Brazil. The packed fruits were placed in portable coolers at a temperature of 5 °C, ensuring they were not stacked to control the transpiration rate and preserve their integrity during laboratory transport. This experiment was conducted using experimental and analytical triplicate to validate the results. Seventy-five berries were selected for analysis, five from each clamshell, characterised by 75% red colouring, uniform size and shape and free of physical or biological damage [27]. The fruits were washed with potable water and disinfected by immersion for 15 min with an effervescent organic sanitiser composed of dichloro-s-triazinetrione sodium and 40.8% active chlorine (Clorin Salad, Rio de Janeiro, Brazil), dissolved in 2 L of water.

Regarding the formulation of the films, 5 treatments by mixing the polymer and glycerol as a plasticiser in different proportions were tested to optimise the mechanical and physical properties of the film (Table 1). The initial ratios were based on the literature [28], which reported the effects of different WPI: glycerol ratios on the mechanical and physical properties, such as strength, flexibility, and moisture resistance of films, which are crucial for a functional coating. In our study, we extended the range of WPI: glycerol proportions tested in the previous survey to achieve improvements in the formulation by adjusting the polymer-to-plasticiser ratios.

### 2.2. Film Preparation

Filmogenic solutions were prepared, with some adaptations, according to the method proposed by Fernandes et al. [29] and Osés et al. [28]. They were prepared by adding distilled water to a mixture of whey protein isolate and glycerol to obtain a solution with a final concentration of 10% (*w*/*w*) total solids. The solution was homogenised at 25 °C for 15 min until complete solubilisation using a T25 Ultra-Turrax homogeniser (IKA, Staufen, Germany). The pH was adjusted to 7.5 using 0.1 mol/L NaOH, through a pH meter model 826 (Metrohm, Herisau, Switzerland). As pH values above the isoelectric point promote excess positive and negative charges, facilitating interactions with water molecules and improving solubilisation [30].

**Figure 1:**
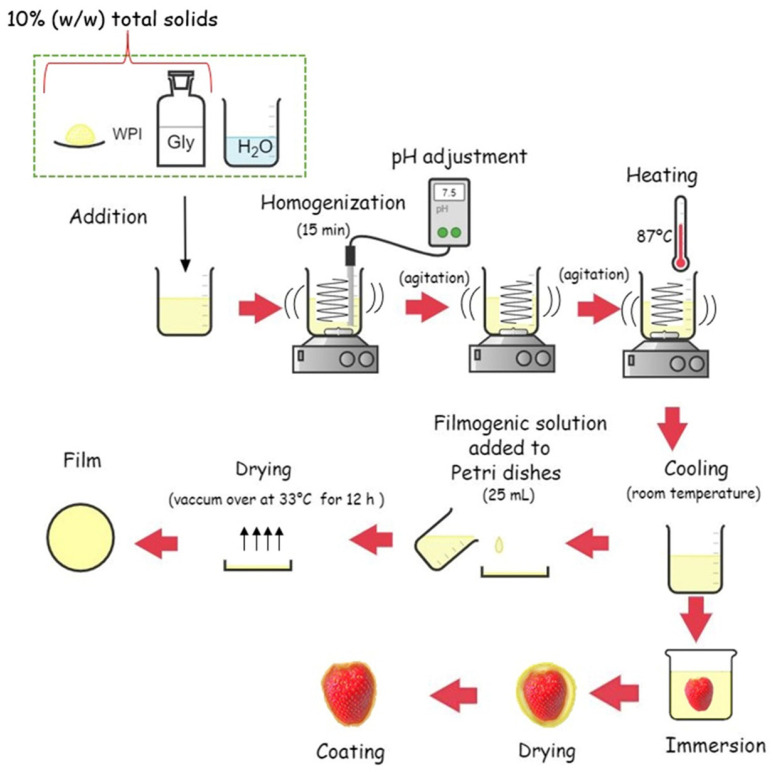
Flow chart of the film production and strawberries coating.

The filmogenic solution was heated with continuous stirring at 87 °C for 20 min in a water bath (WNB 22, Memmert, Germany). After allowing to cool at room temperature, 25 mL of solution was placed in polystyrene Petri dishes with 15 cm diameter and dried at 33 °C in a forced convection incubator model 307 (ELKA, Lüdenscheid, Germany) for 12 h. Subsequently, 15 trays were prepared, each containing 6 strawberries, for a total of 90 strawberries used for analysis. For each tray, 3 strawberries were treated with the filmogenic solution, and 3 were used as controls. Figure 1 shows the sequence of film production and strawberry coating.

### 2.3. Film Characterisation

For the characterisation analyses, the dry films were conditioned in desiccators at 50% relative humidity (using saturated solutions of magnesium nitrate) and 75% relative humidity (using saturated solutions of sodium chloride) and at room temperature (25 °C). The films were placed in sealed containers with the saturated solution at the bottom, ensuring consistent humidity levels. This condition lasted 6 days until the films reached equilibrium moisture content. Afterwards, the analysis was performed. The number of film samples used for characterisation varied depending on the type of analysis performed, with a minimum of three replicates per analysis.

#### 2.3.1. Film Solubility in Water

Film solubility in water (%) was determined according to the method described by Nakashima, Chevalier & Cortez-Vega [31]; the films were cut into pieces (2.0 × 2.0 cm) and dried at 105 °C for 24 h to determine the initial dry mass (*dm_i_*) using an analytical balance (Bioscale, model FA-2204-BI, Sao Paulo, Brazil). Subsequently, these pieces were immersed in 50 mL of distilled water inside a beaker at 150 rpm in an incubator shaker (Solab SL-222, Piracicaba, Brazil) for 24 h at 25 °C. After, the samples were removed and dried in an oven at 105 °C for 24 h, and then the final dry mass (*dm_f_*) was determined using an analytical balance. All analyses were performed in triplicate. The solubility in water (%) was calculated using Equation (1):

(1) $$Solubility\;in\;water\left(\%\right)=\frac{\left({dm}_{i}-{dm}_{f}\right)}{{dm}_{i}}\cdot 100\%$$

#### 2.3.2. Moisture Percentage

The moisture content of the films (%) was determined using the methodology of Costa et al. [32]. The film samples (2.0 × 2.0 cm) were previously weighed on an analytical balance (Bioscale, model FA-2204-BI, São Paulo, Brazil) to determine the initial mass (*m_i_*). Then, they were dried in an oven (Yamato ADP-31, Santa Clara, CA, USA) at 105 °C for 24 h to determine the final mass (*m_f_*). All analyses were performed in triplicate. The moisture was then calculated according to Equation (2):

(2) $$Moisture\left(\%\right)=\frac{\left(m_{i}-m_{f}\right)}{m_{i}}\cdot 100\%$$

#### 2.3.3. Film Thickness

Film thickness was determined using a digital micrometer (Micromar Mahr, Göttingen, Germany) at five different locations: the four vertices and the center of each 2.0 × 2.0 cm film sample. All analyses were performed in triplicate.

#### 2.3.4. Mechanical Properties

Relative humidity plays a significant role in the mechanical behaviour of edible films and coatings; higher RH conditions can affect the water content within the films, influencing their flexibility, tensile strength, and overall stability. Furthermore, strawberries are often stored at varying humidity levels under real-world conditions. Therefore, films performing well at lower and higher RHs can provide more reliable protection for the fruit under different environmental conditions [28]. For this reason, due to practical implications, we investigated the influence of both 50% and 75% RH on the mechanical properties of the films.

Regarding relative humidity, a 75% RH is representative of storage conditions in commercial and domestic cold stores. In addition, many supermarkets and distributors store fruit in environments with RH close to 75% to balance moisture loss and avoid excessive condensation (>85–90%), which favours the growth of fungi such as *Botrytis cinerea* [33]. Thus, since the study’s primary objective is to evaluate the effectiveness of the coating in maintaining visual quality, a moderate RH prevents the effects of condensation from overriding the impact of the coating. Finally, very high RH may mask the ability of the coating to reduce the dehydration of strawberries, as the environment itself would already minimize the water loss from the fruit.

Tensile strength (MPa), elongation break (%), and elastic modulus (MPa) of films were evaluated according to the standard guidelines of the American Society for Testing and Materials [34], using a TA. An XTPlus texture analyser equipped with a 30 kg load cell (Stable Micro Systems, Surrey, UK) has been employed. A total of 20 measurements were performed for each formulation.

Tensile strength was calculated according to Equation (3) [35].

(3) $$\sigma =\frac{F_{max}}{A}$$

where *σ* is Tensile strength (MPa), *F_max_* is the maximum force (N) applied just before film rupture, and *A* is the cross-sectional area of the film (mm^2^).

The elongation break was determined using Equation (4) [36].

(4) $${E(\%)=\frac{L}{D}}_{i}\cdot 100\%$$

where *E* is elongation break (%), *L* is distance at moment of rupture (mm), and *D_i_* is initial grip separation (mm).

Elastic modulus was obtained using Equation (5) [37].

(5) $$Y=\frac{F}{\varepsilon \cdot L}\frac{D_{G}}{A_{x}}$$

where *Y* is the elastic modulus (MPa), F is the maximum force (N), *ε* is the thickness of the film (mm), *L* is the width of the film (mm), *D_G_* is the initial grip separation (mm), and *A_x_* is the elongation at point *x* (mm).

#### 2.3.5. Thermogravimetric Analysis

Thermogravimetric analysis (TGA) was performed using a thermogravimetric analyser (Las Navas Instruments, TGA-2000, Conway, SC, USA), following the method proposed by Bhat et al. [38]. Film samples (approximately 0.5 g) were heated from 25 to 750 °C at a constant heating rate of 10 °C/min. The analyser’s integrated heating system precisely controlled the temperature, ensuring uniform temperature distribution across the sample. A nitrogen gas flow was introduced at a constant flow rate of 20 mL/min throughout the analysis to maintain a controlled inert atmosphere. The flow rate was monitored and regulated to prevent variations in the atmosphere, ensuring consistency and preventing oxidation or other unwanted reactions. All analyses were performed in triplicate.

### 2.4. Rheological Behavior of Filmogenic Solution

Rheological measurements of the filmogenic solution were determined at 4 °C and 25 °C with a rheometer (model MCR Anton Paar GmbH, Graz, Austria) using a CC27 coaxial cylinder apparatus, according to Chakravartula et al. [39]. The measurements were performed at a 10 s^−1^/min shear rate from 3 to 500 s^−1^ using 20 mL of sample. All analyses were performed in triplicate.

The experimental data fitted the Power Law model [40] according to Equation (6):

(6) $$\sigma =K{\left(\gamma ˙\right)}^{n}$$

where *σ* is the shear stress (Pa), *γ̇* is the shear rate (s^−1^), *K* is the consistency index (Pa.s^n^), and *n* is the flow behaviour index (dimensionless).

### 2.5. Strawberries Coating and Packaging

The coating after sanitisation followed the methods proposed by Leite [41]; the sepal and pedicel of strawberries were cut, followed by washing and sanitisation in a 200 mg·L^−1^ sodium dichloroisocyanurate solution for 15 min. Next, the fruits were submerged entirely in the F3 film-forming solution, as previously described, for 1 min at 25 °C and then drained for 2 to 3 min, using plastic screens to eliminate excess solution. The volume used for each solution was 50 mL per 100 g of fruit. The exact volume of solution in each strawberry may vary depending on its size, but the process was designed to ensure uniform coverage. Then, the coated fruits were placed in an incubator chamber (Marconi model MA 403, São Paulo, Brazil) at 4 °C for 17 h to ensure proper coating drying. After drying, fruits were packaged in polyethylene terephthalate (PET) trays and stored at 4 °C in a refrigerator for 8 days, standardising the number of 6 fruits per package. The temperatures selected here address the possible storage temperature (4 °C) and the temperature for applying the coating (25 °C). Brazil is a tropical country, so 25 °C is a suitable temperature for the country’s ambient conditions.

Fifteen PET trays were prepared, with 3 trays for each treatment. Fruits were divided into two groups: T1 (Control)—fruits without coating application, and T2—fruits coated with F3 formulation. Due to its greater mechanical, thermal, and moisture resistance, formulation F3 was selected to coat the strawberries. Thus, 2 treatments were used to evaluate the effect of WPI coverage: a control group and a group coated with F3.

Control strawberries were obtained commercially, transported to the laboratory, disinfected, and stored under the same conditions as the coated strawberries, as previously described. The only particularity of the control strawberries was that they were not immersed in the film-forming solution for 1 min at 25 °C. However, after coating the treated strawberries, both they and the control strawberries were placed on a screen and kept at 4 °C in the incubator chamber, where they remained for 17 h (the time required for the coating of the strawberries treated with the film-forming solution to completely dry). Then, treated and control strawberries were placed in different PET trays and stored at 4 °C in a refrigerator. The quality of both control and coated strawberries was evaluated during storage, as described below.

### 2.6. Mass Loss of Strawberries During Storage

Mass loss (%) was determined by weighing the strawberries in a semi-analytical balance (Ohaus Adventurer ACR 120, Parsippany, NJ, USA) over storage days at 4 °C. The analysis was done on individual fruits placed inside the PET trays. Measurements were recorded at 0, 2, 4, 6, and 8 days. The mass difference was calculated by comparing the mass of the strawberry after coverage and before storage (0) with its relative mass at the subsequent storage days (*n*), according to Equation (7) [42]. That is, the mass of coated strawberries was compared to that of the fresh-coated strawberries. In contrast, the mass of the control strawberries was compared to that of the fresh-control strawberry during the subsequent days of storage, thus calculating the difference in mass for each day of similar fresh product. A statistical comparison of mass loss between the control and coated strawberry groups was conducted across all storage time to determine which group exhibited more pronounced mass loss relative to its respective control.

(7) $$Mass\;loss\;\left(\%\right)n=\frac{\left(m_{0}{-m}_{n}\right)\;}{m_{0}}\;\cdot \;100\%$$

### 2.7. Visual Quality and Shelf Life of Strawberries During Storage

The visual quality of strawberries during the storage period has always been assessed by the same trained personnel (Table 2). They conducted visual quality evaluations using rating scales and descriptors, as described by Kelly et al. [24]. The training was conducted using fresh commercial strawberries and photographs to identify subtle changes in the analyzed attributes. It was led by individuals experienced in applying the rating scales. Colour, shrivelling, and decay severity were determined subjectively using a 1–5 visual rating scale where 5 = excellent, 3 = acceptable, and 1 = poor. Firmness was determined based on the whole fruit’s resistance to slightly applied finger pressure and recorded using a 1–5 tactile rating. Limiting quality factors were established, considering the rating value of 3 as the minimum acceptable quality before the fruit becomes unmarketable, establishing the end of shelf life. Thus, when at least one of the visual quality attributes had attained a rating of 3 or less, the experiment was terminated [25]. The chosen 1–5 scale for subjective analysis of visual quality was selected for its simplicity and ease of use, in addition to having previously been validated for strawberries during storage at 4 °C [24,26].

Subjective quality analysis was conducted independently at each predefined storage time point for both the control and coated groups. Subsequently, the subjective scores for each attribute at each storage point were statistically compared. For any attribute and day showing a significant difference, a higher score indicated superior quality in one group compared to the other.

### 2.8. Statistical Analysis

All analyses were performed in triplicate, and results were expressed as mean ± standard deviation (SD). A one- or two-way ANOVA was used to compare data. When a significant F was found (*p* < 0.05), differences between means were evaluated by Tukey’s multiple tests. For multiple groups with non-normal data, the Kruskal–Wallis test, followed by Dunn’s post hoc test, was employed. Statistical analysis between the two groups was assessed using a two-tailed unpaired *t*-test (*p* < 0.05). Linear regressions were performed with a significance level of 0.05. All statistical analyses were performed with Statistica^®^ version 7.0 (StatSoft Inc., Tulsa, OK, USA). Regarding the visual quality analysis of strawberries, possible sources of bias include subjectivity in ratings and limitations inherent to the evaluation methods currently employed.

## 3. Results and Discussion

### 3.1. Film Appearance

After the last step of film elaboration (drying for 12 h; Figure 1), F1–F3 films were visibly transparent, colourless, flexible, and homogeneous. Their surfaces appeared smooth, without visible pores or cracks. Indeed, WPI films are characterised by brightness, a transparent structure, and a colourless appearance [44], unlike films based on whey protein concentrate (WPC), which exhibit a slightly yellowish colour due to the fat present in the WPC [45]. In addition, WPI films are reported to be significantly more transparent than those based on polysaccharides or blends thereof with WPI [46]. In line with other reports in the literature under similar conditions, the conditioning parameters of the films (such as temperature and pH) did not affect the color, with no evidence of a significant Maillard reaction that could influence the visual perception of color [44,46,47]. However, high opacity values can also be preferred in certain products, such as light-sensitive ones, which provide a protective barrier against light. In these cases, WPI films can be technologically modified by additives that increase their opacity, thus expanding their application possibilities [48].

Regarding glycerol, it is well established in the literature that increasing glycerol concentrations up to 60% reduces the yellowing of WPI films, making them more transparent. This has been previously demonstrated both by instrumental color analysis [45,49] and by human visual perception [50]. Glycerol is a colorless compound, and this effect has been attributed to the dilution of proteins and other colored components in the film-forming solution by glycerol [51]. Moreover, hydrogen bonding interactions among water, glycerol, and whey proteins result in a softer film structure, which affects the optical properties of the films [49]. Accordingly, the films obtained here were visibly transparent and colorless, as illustrated by the formulation containing 30% glycerol (Figure S1). Consequently, the use of glycerol as a plasticizer gives an advantage to WPI-based films, since, according to Yoo & Krochta [46], higher transparency is generally considered desirable for food packaging films and coatings, as consumers prefer to see the food inside.

On the other hand, the formulations F4 and F5 presented cracks in the films. This behaviour can be attributed to the insufficient plasticising effect of the glycerol concentration used. Glycerols are typically employed to prevent cracking by reducing discontinuities and increasing flexibility. However, an anti-plasticiser effect may occur when the glycerol concentration is too low to enhance molecular mobility [52]. Consequently, glycerol concentrations ≤ 20% (Table 1) appear insufficient to achieve the desired plasticising effect when combined with WPI. Because of this, only F1–F3 formulations were subsequently submitted to physical, mechanical, and thermogravimetric analyses.

In addition to appearance, the glycerol level significantly impacts the overall functionality of the film due to glycerol’s role as a plasticiser, which influences the film’s mechanical and barrier properties, as well as the solubility and stability of the film, as discussed below.

### 3.2. Physical Properties of the Films

**Figure 2:**
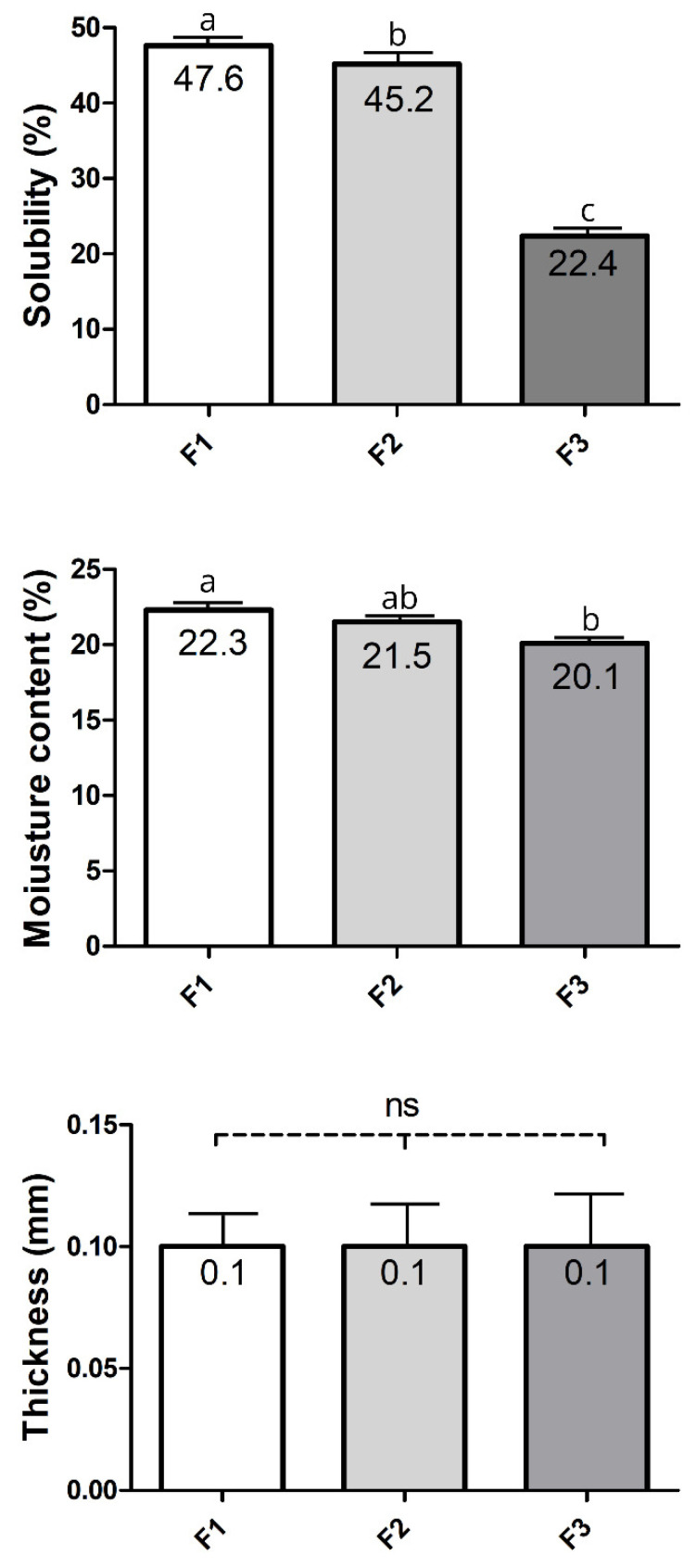
Physical properties of films from different formulations tested at 75% RH. F1: formulation 1 (50% WPI and 50% Gly); F2: formulation 2 (60% WPI and 40% Gly); F3: formulation 3 (70% WPI and 30% Gly); RH: relative humidity; ns: no significant. Analyses were performed in triplicate, and the data are reported as the means ± SD, *p* < 0.05, one-way ANOVA, and post hoc Tukey test: [a, b, c] ≠ among formulations.

The data for physical properties from films with F1–F3 formulations are presented in Figure 2. Solubility is a parameter that indicates the integrity of the film in very humid and aqueous environments [53]. Reduced solubility is essential, particularly when the film is intended to protect high-moisture foods, such as strawberries, with a moisture content of up to 88.26% [54]. This is because films with low water resistance dissolve quickly, leading to an increase in the diffusion of components from the surface to the interior and, consequently, a reduced protective effect on the food surface [55].

The film solubility in water significantly varied among the different formulations tested at 75% RH (Figure 2). In this context, films with lower solubility exhibit greater resistance to dissolution, a favourable effect that contributes to maintaining their structural integrity over time. In line with all films in our study, which have a water solubility of less than 50%, they are considered to have relatively low water solubility, as reported in [52]. This partial insolubility can be attributed to the strong interaction and presence of intermolecular disulfide bonds between protein molecules in the film matrix, resulting from heat treatment [45]. In contrast, the polarity of chitosan, a polysaccharide tested as a potential berry coating, makes it sensitive to moisture, reducing its practical applicability [56]. Also, glycerol influenced the solubility of the films. Increasing glycerol levels from 30% to 50% made the films less resistant to moisture (*p* < 0.05). The solubility behaviour of the WPI: glycerol films observed here is consistent with previous findings of Ramos et al. [45], who developed films using whey protein isolate (WPI) and whey protein concentrate (WPC) with glycerol concentrations ranging from 40% to 60% (*w*/*w*). They also observed that a higher glycerol content promoted better surface wettability of the films, consequently diminishing their moisture resistance due to the hygroscopic nature of glycerol and its plasticizing effect. Glycerol has a hydrophilic character, which allows it to interact with the film matrix, increasing the space between polymeric chains and thus favoring water entry into the film [57].

The WPI: glycerol films developed in this study exhibited lower solubility and greater water resistance than those reported in previous studies. In the study by Ramos, Reinas [45], films based on WPI (10%, *w*/*w*) with glycerol concentrations on the protein base, ranging from 40 to 60%, presented higher values of solubility than those found herein (Figure 2). WPI (5–9%, *w*/*v*) using glycerol as plasticizer (3.6:1, 3:1, and 2:1 of WPI to glycerol) presented 50.58% of solubility by Gounga, Xu [58]. and Fogarasi [59] reported a pronounced solubility of 95% for films with a WPI of 5% (*w*/*w*) and glycerol of 5% (*w*/*w*). Still, low concentrations of WPI (2.0%, *w*/*v*) with glycerol at 40% also led to increased solubility (53.08%) of the films [60]. These findings from the literature suggest a stronger hydrophilic character in previously studied films than those developed in the present study.

It has relevant practical applicability since the solubility of the film can be influenced not only by the intrinsic composition of the film but also by the moisture dynamics of the fruit. Fruits undergo transpiration, accumulating condensed water on their surface, which remains in direct contact with the edible coating [2]. In this context, films with lower solubility exhibit greater resistance to dissolution, a favourable effect that contributes to maintaining their structural integrity over time. In this context, compared to previous research, the lower solubility of the films formulated herein indicates that they can be a more effective protective barrier, reducing shrivelling and limiting microbial contamination while maintaining the fruit’s structural integrity [61,62]. In real-world applications, this means that strawberries coated with such films could experience extended shelf life, particularly in environments with fluctuating humidity. This is particularly relevant for export markets, where fruits undergo long transportation and storage periods.

Variations in WPI concentration, chemical composition, film formulation, and preparation conditions—including plasticisation and pH adjustments—may account for differences in functional properties, such as solubility, in protein-based films [63]. These effects arise due to changes in protein network formation, cross-linking, and interactions with other film components. Higher WPI concentrations, thermal treatment, neutral/alkaline pH, and lower glycerol levels should be considered to achieve lower solubility. Increasing WPI concentrations result in stronger intermolecular interactions (hydrogen bonds, disulfide bonds), creating a denser, more cross-linked structure that is less prone to water dissolving [52]. Regarding pH adjustment, at a neutral to alkaline pH, WPI molecules tend to unfold and expose more reactive groups, such as thiol -SH and amino, leading to more vigorous gel formation and lower solubility [64]. Still, higher thermal treatment (e.g., >80 °C) promotes denaturation and cross-linking, enhancing film stability and reducing solubility [53]. Finally, as for glycerol, higher plasticizer levels increase flexibility but also reduce the density of the protein network, making films more prone to water absorption and dissolution [55]. Therefore, improvement of the film formulation, as proposed in this study (Table 1), is crucial to achieving desirable functional properties.

The moisture content of the films also varied depending on the WPI-to-glycerol ratio (Figure 2). The higher the glycerol content in the formulation, the higher the film’s moisture content will be. These findings corroborated those for solubility in water (Figure 2). A similar behavior for moisture content, considering the WPI: glycerol ratio, was previously reported [28]. Glycerol has a hygroscopic character, which favors the moistening of the film surface and moisture absorption [45,65]. Osés et al. [28] observed that WPI-films (10%, *w*/*w*) plasticised with glycerol at 30, 40 or 50% had moisture content ranging from 21 to 27.5%, which were higher values than those reported for the films herein (Figure 2). In contrast, Ramos et al. [64] reported lower values of moisture (15.10% to 16.82%) for WPI (10%, *w*/*w*) films with 40 or 50% of glycerol content. The differences in moisture content of the films in studies with similar concentrations of WPI and glycerol employed here can be explained by the physicochemical distinctions in their preparation conditions [63], as previously discussed in the present study. In addition, the surface of chitosan–whey protein films richer in protein has been described to be more hydrophobic than that richer in chitosan [66]. This broadens the discussion by highlighting the more hydrophobic nature of WPI films compared to those containing other water-soluble polysaccharides.

The thicker the film, the greater its opacity and puncture force [67]. Furthermore, a high film thickness produces an increase in water vapour pressure (WVP). Therefore, control of this parameter is crucial. However, no differences (*p* > 0.05) were found for this parameter herein (Figure 2). Indeed, the WPI: glycerol ratio is generally reported not to affect film thickness. At the same time, the polymer concentration (WPI) has a significant role in modifying this parameter [58]. In line with this, a similar thickness was found for films with the same WPI concentration and WPI: glycerol ratio as in the present study [65].

### 3.3. Mechanical Properties of the Films

**Figure 3:**
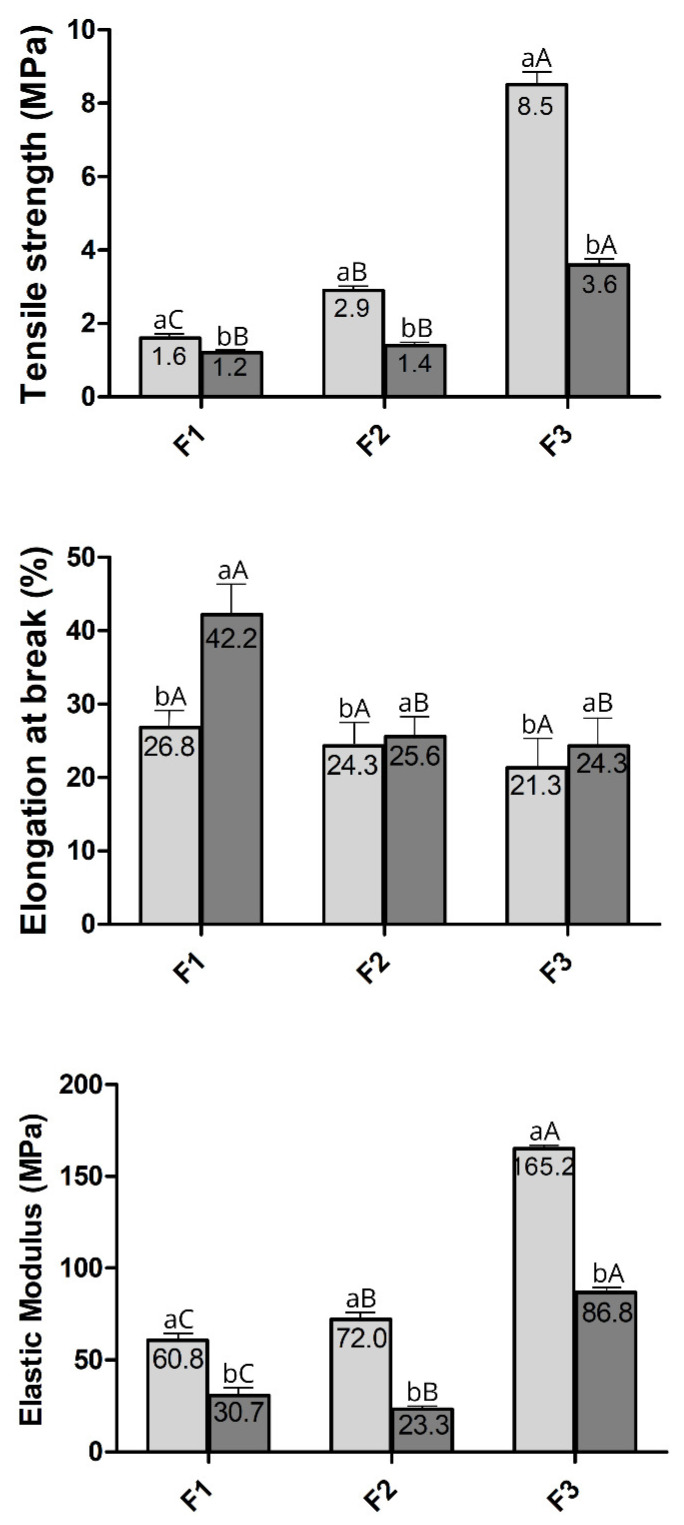
Mechanical properties of films from different formulations tested at 50% RH (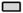) and 75% RH (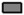). F1: formulation 1 (50% WPI and 50% Gly); F2: formulation 2 (60% WPI and 40% Gly); F3: formulation 3 (70% WPI and 30% Gly). Analyses were performed in triplicate, and the data are reported as the means ± SD. Two-way ANOVA followed by Tukey’s post hoc test, *p* < 0.05; a, b Means in the columns with different superscripts indicate significant differences among relative humidity. A, B, C Means in the columns with different superscripts indicate significant differences between formulations.

The effects of different WPI: glycerol ratios on the mechanical properties of the films were summarised in Figure 3. Only F1, F2, and F3 were selected for mechanical property analysis as they remained intact after drying. This is crucial since films used as packaging or as a complement to synthetic packaging must act as a barrier between the internal and external environment, ensuring strength and integrity to preserve the food. Tensile strength (TS) is the maximum strength a film can withstand against an applied tensile stress, while the elongation at break (EB) represents the ability of a film to stretch [68]. TS and EB are key indexes reflecting the film’s mechanical properties since they are usually related to the film network microstructure and the intermolecular force [69].

Due to the influence of relative humidity (RH) levels on the mechanical properties of films [28], two RHs (50% and 75%) were tested. Regardless of the RH level, the increased WPI-to-glycerol ratio in the formulations raised the tensile strength (TS) (*p* < 0.05), indicating more resistant films to tensile stress (Figure 3). This can be attributed to the higher protein concentration, which promotes the formation of additional S-S covalent bonds (disulfide bridges) among sulfhydryl groups in proteins within the film matrix, increasing its structural stiffness [53]. Furthermore, the TS values showed an inverse relationship with RH content; the 75% RH level resulted in lower TS values than the 50% RH level. This reduction in mechanical strength associated with increased RH is likely due to the plasticising effect of absorbed moisture, which alters intermolecular interactions, increases chain mobility, and reduces film stiffness [70,71].

Unlike TS, EB was reduced by an increased WPI: glycerol ratio at 75% RH. This trend was also observed at 50% RH, although it was not significant (Figure 3). Since EB measures the film’s ability to stretch before breaking (indicative of flexibility), an increase in TS is typically associated with a decrease in EB since more tensile-resistant films tend to be less elastic, demonstrating a typical inverse relationship between tensile strength and elongation at break in polymer films [72]. In line with this, decreasing the glycerol (plasticizer) concentration reduces the magnitude of the film’s elongation [73]. The glycerol reduces the density of protein–protein interactions, leading to an increase in the mobility of polypeptide chains, which results in less resistant but more elastic films [74].

Elastic modulus (EM) indicates the material’s stiffness, which is the film’s resistance to elastic deformation before rupture, and is estimated through the relationship between TS and EB. The larger the module, the more rigid the film is [36]. Regardless of the RH, EM values increased with the WPI: glycerol ratio (Figure 3). In line with this, the formulation F3 produced stiffer films (*p* < 0.05). Generally, an increase in TS is associated with a higher EM, as a film more resistant to tension also tends to be more rigid [37]. This agrees with the findings here for TS (Figure 3). The increase in EM values observed in formulation F3 (70% whey protein, 30% glycerol) can be attributed to several key factors. The higher concentration of whey protein can promote a more organised protein network with stronger intermolecular interactions, increasing the rigidity of the film. The reduced glycerol content limited the plasticisation of the material, which can enhance its vitreosity and molecular order, leading to a more compact and rigid network [75,76]. These changes resulted in an improved TS: EB ratio, as the film became stronger but less flexible [53,74].

Under similar RH conditions and WPI: glycerol ratios, the films used in the present study were stiffer and less flexible than those previously reported by Ramos et al. [45]. Since we heated the film-forming solution to a higher temperature, this explains, at least partially, the greater rigidity of our films compared to the other [45]. The heating likely promoted more significant protein denaturation and intermolecular interactions. Heat treatment is essential for forming intermolecular bonds, thereby contributing to the development of a cross-linked and more rigid polymeric network [77]. Conversely, the films produced in this study were less rigid than those made by Osés et al. [28], even under similar conditions and WPI: glycerol film formulations. Although the heating temperature was similar, the shorter exposure time used in our study may have limited the development of a more robust polymeric structure. Generally, a packaging film requires adequate mechanical strength and extensibility [78]. However, the suitability of the mechanical properties of the films depends on their application. Thus, the temperature and heating time of film-forming solutions should be considered and adjusted according to the intended use of the film. Adequate mechanical strength ensures the integrity of a film. Stiffer, less flexible films improve mechanical resistance and protection against physical damage, accommodating handling and transportation stresses. This makes them suitable for application on delicate foods, such as fresh berries [79]. In contrast, lower stiffness and higher flexibility are more appropriate for food systems where the films should break up during the cooking or mastication process [66]. In addition, they offer sufficient flexibility to adapt better to foods with irregular surfaces, thereby improving sealing and making them more suitable as coatings that conform to the food surface [80].

The tensile strength (TS) of the stored films decreased as the relative humidity (RH) of the environment increased (*p* < 0.05) across all tested formulations (Figure 3). This trend was further supported by the elastic modulus (EM) values, which also showed a significant reduction with rising humidity (Figure 3). A similar pattern has been previously observed in whey protein isolate (WPI) films plasticized with glycerol, where TS and EM decreased as RH increased from 50% to 75% [28].

Conversely, higher RH levels led to an increase in elongation at break (EB) for all formulations tested (*p* < 0.05), as shown in Figure 3. This aligns with prior findings for WPI films plasticized with glycerol and sorbitol, which exhibited greater EB at 75% RH compared to 50% RH [28]. This effect is attributed to water’s plasticizing action, which reduces stiffness and strength while enhancing the film’s flexibility [71].

### 3.4. Thermogravimetric Analysis of the Films

**Figure 4:**
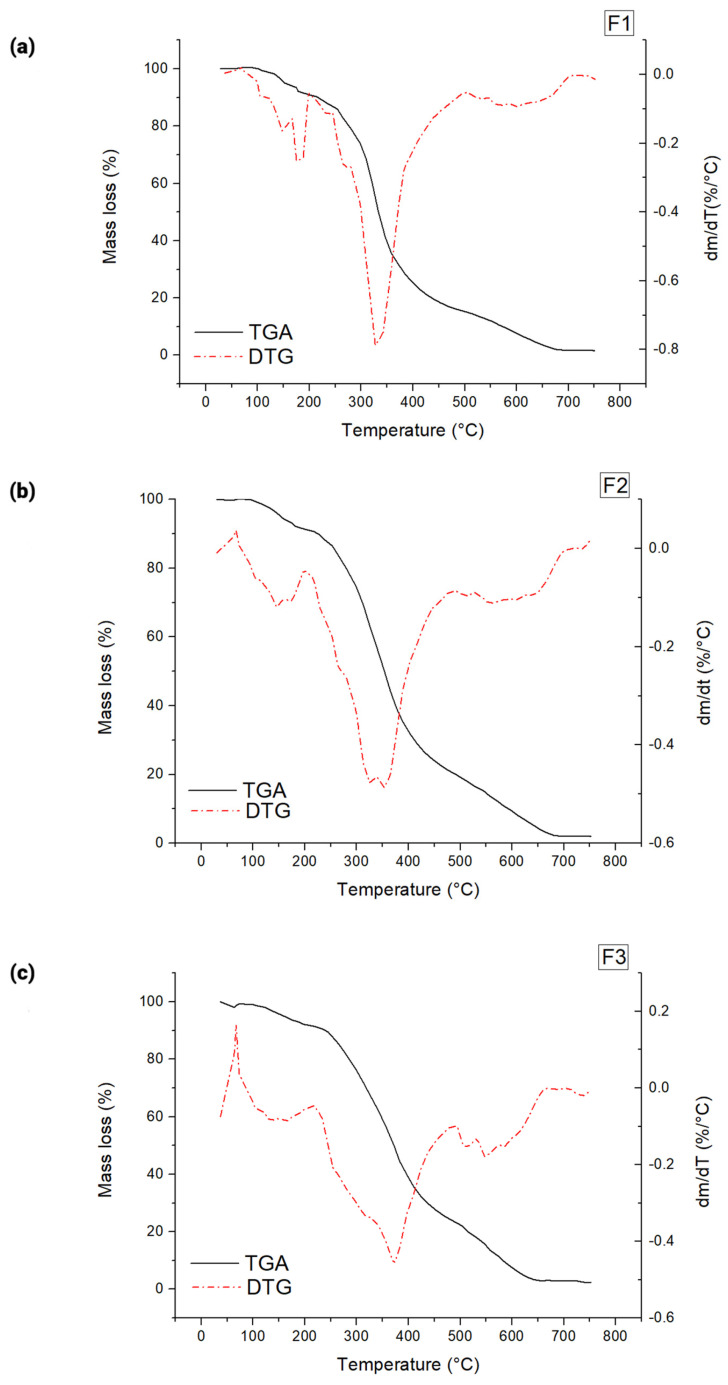
Representative derivative thermogravimetry (DTG) curves of whey protein isolate (WPI) films: (**a**) F1, (**b**) F2, and (**c**) F3. F1: formulation 1 (50% WPI and 50% Gly), F2: formulation 2 (60% WPI and 40% Gly) and F3: formulation 3 (70% WPI and 30% Gly). dm/dT: first derivative of the variation in mass as a function of temperature.

The thermal degradation of polymers results in the breaking of bonds due to heat in the absence of oxygen [81]. Thermogravimetric (TGA) profiles of the films from different WPI: glycerol ratios were evaluated for thermal stability. The DTG curve helps determine the number of decomposition stages by analysing the mass loss rate with temperature. Three events in mass loss occurred in all formulations investigated (Figure 4). The first stage was observed between 67 and 200 °C, corresponding to water loss [81]. The second mass loss was observed in the range of 200 to 500 °C, associated with the degradation of proteins and the amount of glycerol present in the formulation [82]. The third decomposition stage occurred at approximately 500 to 710 °C, resulting from the degradation of carbon residue formed during the second stage [83]. These results are consistent with the thermal degradation of edible WPI: glycerol films previously reported by others [81].

The first stage corresponds to the evaporation of residual moisture within the film [72], which is crucial for maintaining its flexibility and mechanical properties. Since this stage was between 67 and 200 °C, this means that exposure to moderate heat may cause dehydration, leading to increased brittleness and reduced film integrity [84]. Thus, the film may become more brittle when exposed to moderate temperatures, such as during drying, mild heating, or prolonged storage in warm conditions. The second stage leads to the thermal degradation of whey protein and glycerol, compromising the film’s integrity [73]. Since this stage occurred between 200 and 500 °C, this suggests that the film is unsuitable for high-temperature applications, such as baking, frying, or sterilisation processes. Thus, the film formulated here is ideal for applications such as fresh packaging or moisture-sensitive food products [84]. Additionally, the film should not be subjected to temperatures above 200 °C, which limits its use in milder thermal food processing methods, such as hot filling or pasteurisation. The third stage is beyond the temperature range of most food-related applications. However, it is relevant for understanding the film’s complete thermal decomposition profile, which can be helpful in considering biodegradability and waste management [85].

The temperatures for the onset of maximum degradation (T_max_), taken from the peak temperature of derivative weight % curves, are listed in Table 2. The thermal stability decreased as the WPI: glycerol ratio was reduced from 60% to 40% (*w*/*w*) for WPI in the films. The arrangement of glycerol molecules between the polymer chains reduces the cross-linking density between the whey proteins, consequently reducing the thermal stability of the films [82]. Indeed, a higher glycerol level resulted in a lower T_max_ (*p* < 0.05), indicating a decrease in the film’s thermal resistance. Inline, glycerol also decreased (*p* < 0.05) the temperature of initial decomposition (T_d_) of the films, which can be attributed to its mass loss through evaporation [86]. Still, decreasing glycerol concentrations significantly delayed the mass loss at temperatures above 300 °C (Table 3; Figure S2). This finding corroborated greater stability of films with lower glycerol levels in the thermal decomposition [87].

Films, both with higher and lower thermal stability than that found here, have been previously reported for films with similar WPI: glycerol ratios. Ramos, Reinas [45] obtained higher values of T_d_ and T_m_ and consequent lower values of mass loss. In contrast, Kadam, Thunga [81] found lower T_m_ than that reported herein (Table 3). The differences between our and other studies can be attributed to different conditions of formulation procedures and conditioning of films, such as the final pH of film-forming solutions and RH of drying and storage of films, due to their impact on the protein network structure, cross-linking, and molecular interactions. A neutral to alkaline pH (>6.5) promotes stronger cross-linking (via disulfide bonds and hydrophobic interactions), resulting in a more thermally stable film with higher T_d_ and T_m_ [88]. Higher relative humidity (RH) during drying/storage can lead to higher water content in the film, acting as a plasticizer, which lowers T_d_ and T_m_ by reducing intermolecular forces and making the protein matrix more flexible [89].

The thermogravimetric analysis (TGA) in this research showed that higher temperatures sped the decomposition of whey protein films, reducing their mechanical properties through moisture loss and glycerol volatilisation. Thus, preserving the integrity of the films depends on storing them at 4 °C, which delays these effects and extends the shelf life of enclosed goods. This emphasises how essential it is to maintain the function of these films in food packaging uses by managing the storage environment.

### 3.5. Application of the Film-Forming Solution as an Edible Coating for Strawberries

As the F3 film-forming solution exhibited greater mechanical, thermal, and moisture resistance, it was assessed for its rheological behavior and functional performance as a strawberry coating during storage. The temperatures tested here were chosen to cover the possible storage temperature (4 °C) and the coating application temperature (25 °C).

#### 3.5.1. Rheological Behaviour of the Film-Forming Solution Applied to Strawberries

The Power Law model suitably fits the rheological properties of the film-forming solutions F3 (70% protein, 30% glycerol), as demonstrated by the high determination coefficients of 0.999 (Table 4; Figure S3). This formulation was selected due to its superior film-forming physical and mechanical properties, making it an ideal candidate for evaluating the rheological behaviour.

The consistency index (*K*) is an indirect indicator of the fluid’s viscosity. Therefore, the filmogenic solution was more viscous at 4 °C (higher *K* values) than at 25 °C (lower *K* values) *(p* < 0.05). These results were corroborated by the higher slope of the curve at 4 °C (Figure S3). An inverse relation between viscosity and temperature was previously reported for WPI films [90]; decreasing temperature enhances viscosity due to reduced intermolecular distances [53]. Coherently, the values of shear stress for the solution were highest at 4 °C (Figure S3); thereby, the solution is more resistant to deformation at a colder temperature than at 25 °C. As the viscosity increases, fractional free volume decreases; consequently, the space between the polymeric chains decreases [90].

The inverse relationship between viscosity and temperature has important implications for practical applications. Higher viscosity (higher *K* values) at storage temperature indicates that the solution is thicker and more resistant to flow, which reduces the risk of excessive dripping or uneven distribution of the previously applied coating in the stored product [91]. This is due to the decrease in particle mobility in the solution and the maintenance of adherence to the coating [46]. Conversely, lower viscosity during application contributes to a more straightforward application of the coating and ensures adequate particle mobility for homogeneous coating of the fruit surface [91]. However, lower viscosity may result in thinner coatings. Therefore, emerging studies have investigated the effect of applying polymeric multiple coating layers to ensure their effectiveness as a protective barrier [92]. Nevertheless, it is essential to highlight that a greater coating thickness reduces the sensory scores for taste [93]. Furthermore, this behavior illustrates the relevance of controlling the storage temperature to adequately maintain the functional barrier properties, which are enhanced by the higher viscosity. In this scenario, our findings suggest immersing the fruit in the filmogenic solution at 25 °C for 1 min, followed by storage at 4 °C. This process helps decrease the mobility of the particles in the solution and ensures better adherence of the coating at storage temperature, in line with previous studies on polymeric coatings [94,95].

At similar temperatures, the *K* values for film-forming solutions from the literature were much higher than those obtained in the present study. The higher WPI concentrations used by Sevim [96] and the increased WPI: glycerol ratios justify the higher viscosity, as more polymeric crosslinks are formed. Still, there is a superior viscosity of polysaccharide-based filmogenic solutions compared to protein solutions [97]. However, although a highly viscous coating provides a more efficient barrier, it lowers the taste perception in polymer suspensions for food applications. This is because a high viscosity can hinder the diffusion and release of flavour compounds, reducing their interaction with taste receptors on the tongue [93]. Indeed, coatings with 1% chitosan were considered to diminish the overall sensory quality when compared with uncoated strawberries [98].

The flow behaviour index (*n*) indicates the degree of non-Newtonian characteristics of the fluid: When “*n*” is 1, the fluid is Newtonian; if “*n*” is > 1, the fluid is shear thickening; and if 0 < “*n*” < 1, the fluid is shear thinning [99]. Thus, the film-forming solution exhibited Newtonian behaviour at both temperatures tested (Table 4). The linearity between shear stress and shear rate (Figure S3) corroborated the solution’s Newtonian behaviour [100]. Consistent with these findings, WPI (8 to 11%, *w*/*w*) glycerol solutions have been described as exhibiting Newtonian behavior or a shear-thinning character at higher concentrations of WPI [96]. Finally, the temperature did not change the type of flow of samples (*p* > 0.05). Indeed, differences in flow behavior are more dramatic for different WPI concentrations and, to a lesser extent, for differences in temperature [96].

#### 3.5.2. Mass Loss of the Coated Strawberries During Cold Storage

**Figure 5:**
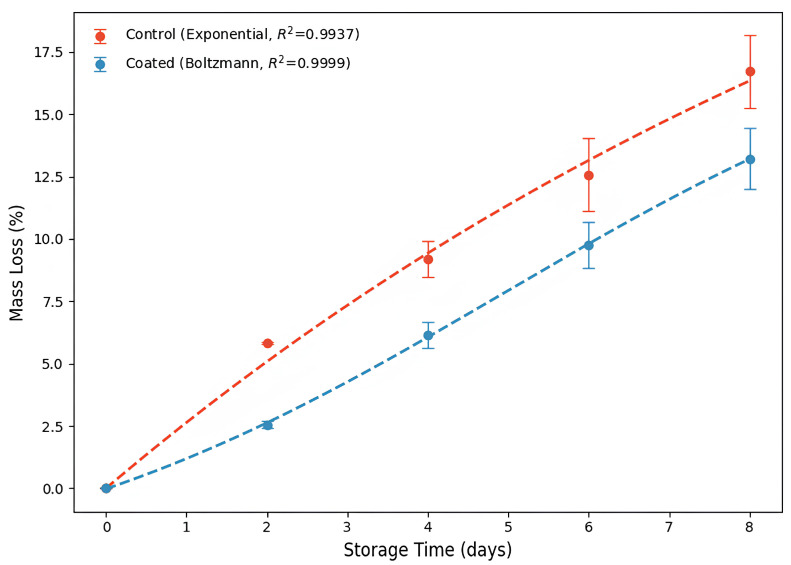
Analysis of the strawberries’ mass loss (%) values over storage days at 4 °C and 75% RH.

Although strawberries should be stored at 0 °C to maximize shelf life, our study focuses on commercial quality and application. Thus, 4 °C simulates the conditions of a household refrigerator. Similarly, the discussion in our article highlights the testing of edible polymer coatings in the literature, with temperatures ranging from 4 °C to even room temperature to assess the practical applicability of the coatings [4,41]. Regarding relative humidity, a 75% RH is representative of storage conditions in commercial and domestic cold stores [33]. Thus, the preservation potential of the edible coating on strawberries was evaluated under controlled conditions of humidity (75% RH) and temperature (4 °C), as shown in Figure 5.

Respiration and moisture evaporation through the skin are the primary factors contributing to the loss of mass in strawberries [33]. Coating preserved the strawberries’ mass during storage by an average of 3.16% compared to the control non-coated fruit (*p* < 0.05) (Table S1). The greater slope of the curve for control strawberries illustrates the most significant mass loss (Figure 5). Nonpolar amino acid residues and intermolecular disulfide bonds enable the WPI-coating to act as a substantial barrier to water vapor, thereby mitigating moisture loss in fruit [45]. Despite the smaller magnitude, coating strawberries also reduced (*p* < 0.05) their mass during storage (Table S1). Similar behavior was previously reported for mass loss in control and chitosan-WPI conjugate-coated strawberries during 8 days of storage at 5 °C and 75% RH [4].

Different mechanisms have been proposed for the ability of WPI films to reduce moisture loss. By forming a dense and uniform physical barrier on the surface of the strawberry, it minimises the direct exposure of the fruit to air, reducing the rate of moisture evaporation from the fruit’s surface. However, efficiency depends on the thickness and uniformity of the coating [101]. In addition, WPI forms a matrix of protein chains closely joined by cross-linking, which reduces water vapor permeability by mitigating the migration of water molecules from the interior to the surface of the fruit [102]. WPI also increases the water retention capacity of the fruit by interacting with water molecules through hydrogen bonding [103]. Interestingly, recent papers have demonstrated the ability of WPI mulch to adjust to environmental humidity, which has also been suggested to contribute to mitigating water loss from the fruit [5].

#### 3.5.3. Visual Quality Evaluation of the Strawberries

The perishability of strawberries makes visual quality a crucial factor in consumer perception and marketability. Indeed, market studies show that consumers are willing to pay a premium price for strawberries with better visual appeal, colour, and texture [104]. Colour, brightness, texture, and shrivelling are key visual quality factors influencing consumer preferences and purchasing decisions. The colour is a primary quality indicator; bright red hues are associated with optimal ripeness, freshness, and sweetness, while darker or dull-coloured strawberries are often perceived as overripe or deteriorated [105]. The surface appearance and brightness contribute to the perception of freshness [106]. Consumers also assess the freshness of strawberries based on texture and firmness; softer strawberries indicate overripeness and microbial spoilage, leading to rejection at the point of sale [107]. Consistently, poor texture, lack of juiciness, and reduced flavour are associated with shrivelling strawberries by consumers [33]. Given the significant influence of visual quality on consumer acceptability and marketability, it is crucial to assess how the applied coatings impact the strawberries over time.

**Figure 6:**
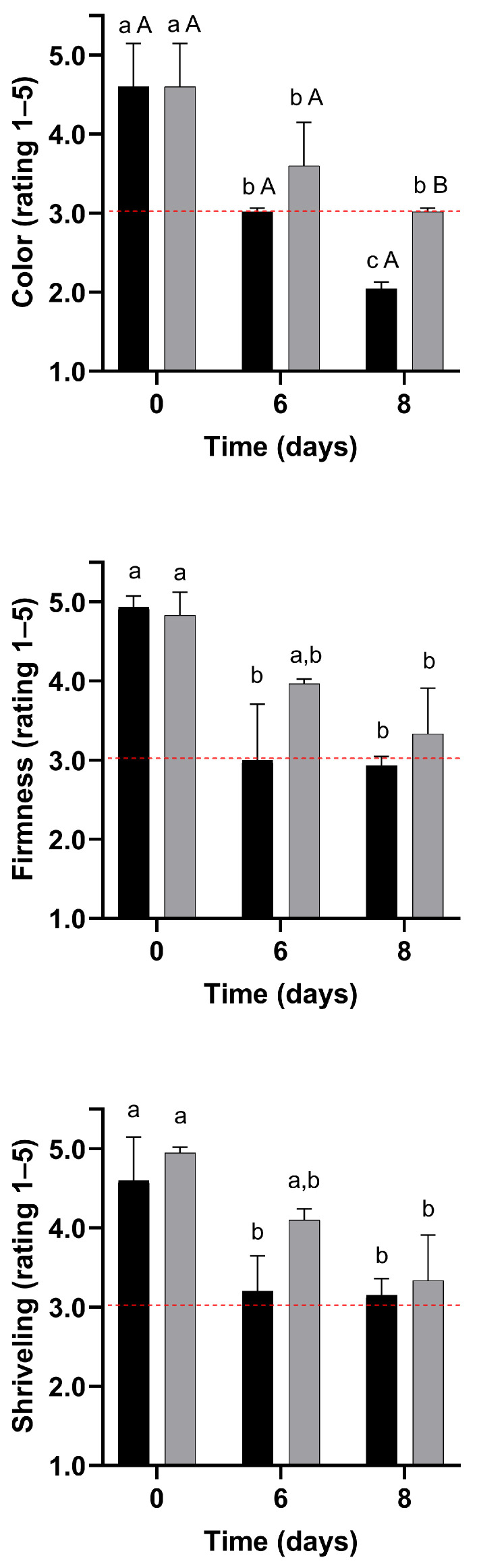
Colour, firmness, and shrivelling ratings for strawberries untreated (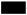) or coated with F3 film-forming solution (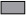) after storage for 8 days at 4 °C and 75% relative humidity. 5 = Excellent; 3 = Acceptable; 1 = Very poor. Dash lines represent the minimum acceptable for sale (rating of 3). Analyses were performed in triplicate, and the data are reported as the means ± SD. Two-way ANOVA followed by Tukey’s post hoc test, *p* < 0.05; a, b, c Means in the columns with different superscripts indicate significant differences among days of storage. A, B Means in the columns with different superscripts indicate significant differences between treatments.

**Figure 7:**
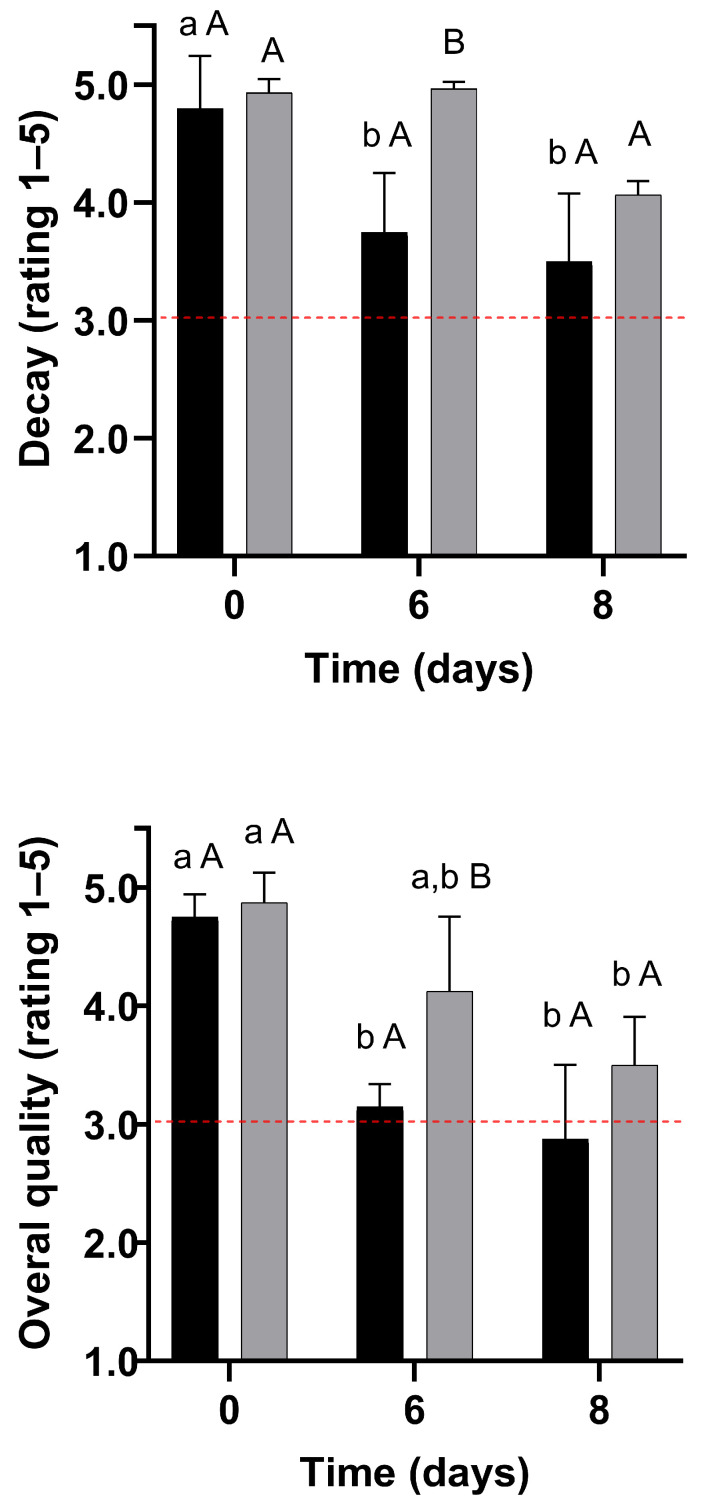
Decay and overall subjective quality ratings for strawberries untreated (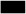) or coated with F3 film-forming solution (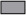) after storage for 8 days at 4 °C and 75% relative humidity. 5 = Excellent; 3 = Acceptable; 1 = Very poor. Dash lines represent the minimum acceptable for sale (rating of 3). Analyses were performed in triplicate, and the data are reported as the means ± SD. Two-way ANOVA followed by Tukey’s post hoc test, *p* < 0.05; a, b Means in the columns with different superscripts indicate significant differences among days of storage. A, B Means in the columns with different superscripts indicate significant differences between treatments.

In this scenario, visual quality evaluation ratings for untreated and WPI-coated strawberries are shown in Figure 6 and Figure 7. The formulation F3 has been selected for its enhanced mechanical properties.

##### Color

**Figure 8:**
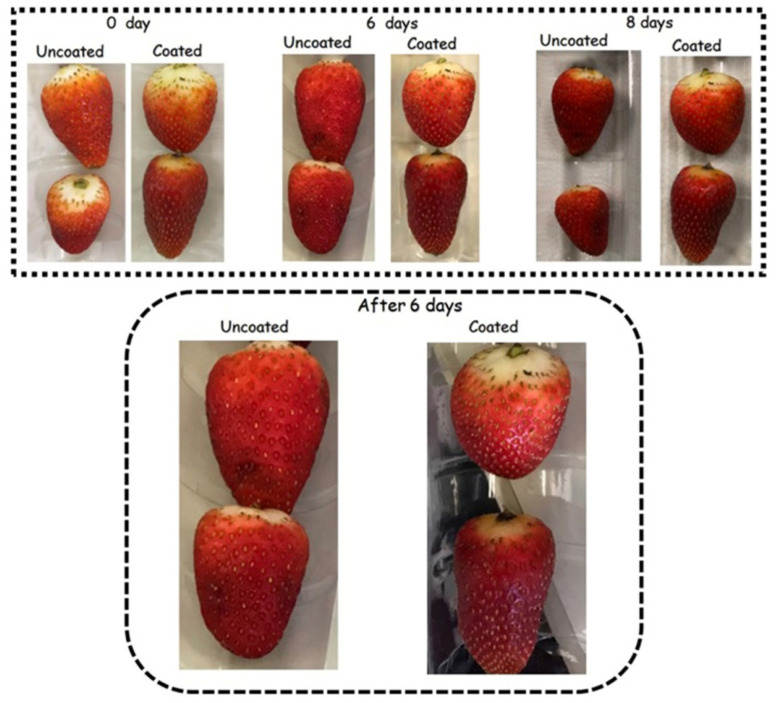
Effect of biodegradable coating on the preservation of strawberries during 8 days of refrigerated storage (4 °C; 75% RH).

During storage, colour ratings were reduced by up to 56% and 34% in the control and coated samples, respectively (*p* < 0.05). The colour of coated strawberries was better preserved than that of fruit from the control treatment (Figure 6). Consequently, untreated strawberries became unacceptable for sale after 6 days of storage, while coated strawberries reached this threshold only on day 8. Indeed, visible darkened areas started to appear on the surface of control strawberries after 6 days of storage. In contrast, these spots only emerged on day 8 for the strawberries coated with the edible coating (Figure 8). Thus, treatment with WPI reduced visual colour deterioration in strawberries (*p* < 0.05).

Water loss during storage is a key factor contributing to shrivelling in strawberries, which in turn causes shrivelled and darkened spots on the fruit’s surface [108]. Thus, the significant delay in water loss provided by the coating (Figure 5) may have contributed to preserving the colour of the coated strawberries during storage.

##### Shrivelling and Firmness

During storage, firmness ratings were reduced (*p* < 0.05) by up to 41% and 31% in control and coated strawberries, respectively (Figure 5). Due to the smaller decrease in firmness ratings, the firmness of WPI-coated strawberries remained acceptable for sale, whereas the control samples became unacceptable within 6 days. Coated strawberries tended to have higher shrivelling ratings, although this difference was not statistically significant (*p* > 0.05). Indeed, visible darkened areas associated with shrivelling appeared on the surface of control strawberries after 6 days of storage, whereas they only emerged on the coated strawberries after 8 days (Figure 8). The WPI-coating forms a hydrophobic barrier that mitigates water loss and prevents fruit shrivelling and texture changes [45]. Mitigation of water loss, shrivelling, and softening in coated strawberries with different polymeric matrices has been extensively reported in the literature during refrigerated storage [109,110,111,112,113,114,115,116].

##### Decay

Decay ratings remained stable for the coated strawberries throughout storage. In contrast, decay increased by up to 27% for the control strawberries; however, they never attained objectionable levels during storage (Figure 7). Nonetheless, WPI-coated strawberries showed up to 32% less decay than untreated strawberries stored for 8 days at 4 °C. The thin skin of strawberries facilitates water loss, physical damage, and microbial contamination during storage, leading to deterioration [33]. In contrast, the WPI coating improves the mechanical barrier properties, which may protect the fruit against physical damage and fungal decay, as suggested by previous studies [4].

##### Overall Quality

The overall quality of strawberries decreased during storage (*p* < 0.05) by up to 39% and 28% for control and coated samples, respectively. The reduction in the overall quality of strawberries can be attributed to the decrease in colour, shrivelling, firmness, and decay ratings (Figure 6 and Figure 7). Due to the smaller magnitude of the overall quality decrease during storage, WPI-coated strawberries showed up to 31% higher overall quality than the untreated strawberries. Consequently, compared to the control, the quality of WPI-coated strawberries remained acceptable during 8 days of storage at 4 °C (Figure 7).

#### 3.5.4. Shelf Life of the Strawberries

Considering a quality rating of 3 as the minimum acceptable quality for sale, colour, firmness, and overall quality were limiting quality factors for the control strawberries (Figure 6 and Figure 7) [25]. Therefore, the strawberries from the control treatments were considered unacceptable for sale due to objectionable colour and firmness within 6 days of storage at 4 °C, establishing the end of shelf life. In contrast, colour was the limiting quality factor for coated strawberries, making them unmarketable on day 8 of storage. Therefore, the coating led to a 33% shelf life extension in strawberries stored at 4 °C and 75% RH.

Photographs of strawberries illustrate how the coating extended their shelf life during storage (Figure 8). The extended shelf life of strawberries, as achieved with the WPI coating developed herein, can have significant economic and marketability impacts. Strawberries are highly perishable fruits, with postharvest losses, mainly due to deterioration during transportation and storage, being a primary concern for producers and distributors. By extending shelf life, coatings like WPI can mitigate primary causes of spoilage, increasing the marketability of the product. Additionally, WPI-based coatings, derived from natural sources and a byproduct of the dairy industry, may appeal to consumers seeking eco-friendly and sustainable options, further enhancing their market potential [5].

Regarding the distribution chains, strawberries with an extended shelf life can be stored and transported for extended periods before reaching consumers. As a result, the frequency of restocking and logistical costs can be reduced [117]. Moreover, a longer shelf life ensures that strawberries remain fresh throughout shipping, making them viable for international markets and opening new revenue streams for producers. [118].

Although the strawberries used in our study were purchased from a supermarket, with no control over the time since harvest or the storage conditions before sale, our focus is on the shelf life extension, in days, of strawberries coated with the polymeric film compared to the uncoated control. While initial conditions may vary between studies, the basis of comparison in our case was focused on the improvement in the shelf life of the coated strawberries, which provides a valid basis for comparisons among different studies, as it evaluates the effect of the coating on extending the product’s quality during storage. Therefore, for the comparative purposes among studies of this section, the term ‘shelf life extension’ refers to strawberries coated with a polymeric coating, compared to the uncoated control for each respective study.

Studies evaluating the isolated effect of WPI on the shelf life extension of strawberries were not found in the literature. However, a coating of chitosan-WPI conjugate with glycerol as a plasticiser, in conditions similar to those tested here, extended the shelf life of strawberries by 3 days compared to the uncoated control (from 5 to 8 days of storage) at 5 °C and 75% RH [4], while in the present study, the extension was 2 days. It is essential to highlight that the combination of polymers in the coating has been reported to present a synergistic effect on the shelf life extension of fruits [119]. In line, when chitosan alone (1%) was tested without the use of coating enhancers and without a plasticizer, it extended the shelf life by 2 days compared to the uncoated control (from 4 days to 6 days) for strawberries stored at 10 °C and 90% RH. In contrast, when chitosan is combined with monomethyl fumaric acid, an antimicrobial and antioxidant, the shelf life is extended by 4 days compared to the control (from 4 to 8 days) under similar storage conditions [120]. Similarly, another study reported that early signs of mould development appeared at 8 days of storage at 13 °C and 95% RH for strawberries coated with both 1% and 1.5% chitosan without enhancers [121].

Thus, considering the comparable shelf life extension observed in this study and reports in the literature for chitosan-based coatings without enhancers, alongside the promising results for water loss control, WPI demonstrates potential as a coating for preserving strawberries during refrigerated storage. However, despite achieving similar preservation outcomes, WPI lacks the antimicrobial properties attributed to chitosan, with both polymers sharing only antioxidant activity [13,122]. Therefore, it can be hypothesised that incorporating enhancers, such as antimicrobial and bioactive compounds, into WPI-based coatings could amplify their effectiveness, potentially achieving or even surpassing the conservation performance of chitosan, which remains the predominant polymer studied for strawberry preservation [123].

Among biodegradable polymers, a recent study demonstrated that silk fibroin protein coatings extended the shelf life of strawberries from 3 to 8 days—an increase of 5 days—compared to the control. Silk fibroin effectively preserved strawberries at room temperature (22 °C; 38% RH), an ability rarely observed in other polymers [124]. This effect has been attributed to its antimicrobial and antioxidant properties, as well as its superior barrier function compared to conventional polymeric coatings, which tend to be more permeable. When processed in an aqueous solution, silk fibroin undergoes conformational reorganisation, forming lamellar structures rich in β-sheets. This structural transition increases matrix crystallinity, thereby enhancing resistance to gas and moisture permeability [125] and contributing to the prolonged preservation of strawberries, even at room temperature. However, despite its promising functional properties, no studies have evaluated its sensory acceptability in food applications, highlighting a gap in the literature regarding its practical feasibility as an edible coating.

This discussion emphasises the need to explore other biodegradable edible polymers to advance the field of berry preservation, which currently focuses primarily on chitosan and secondarily on plant-derived proteins. Regarding chitosan, recent studies have shifted towards enhancing these coatings by incorporating polymer nanoencapsulation, conjugating them with antimicrobial bioactive compounds or additives, or combining them with a modified atmosphere packaging to amplify their effectiveness [126]. In this sense, edible coatings using chitosan nanoparticles and glycerol as a plasticizer increased the shelf life by 8 days (from 8 to 16 days) for strawberries stored at 6 °C [127]. Polymer nanoencapsulation enhances the functional properties of the coating, thereby extending its shelf life through various mechanisms of action. Nanoparticles exhibit a larger surface area-to-volume ratio, which enhances their interaction with microbial cell membranes and consequently potentiates the antimicrobial effect of compounds such as chitosan [128]. Additionally, the increased surface-to-volume ratio yields a more uniform and cohesive coating, which more efficiently reduces gas exchange and moisture loss [129].

Regarding polymeric coatings combined with bioactive compounds, coatings based on the polymer carboxymethyl cellulose, with glycerol as a plasticiser, and a microbial control agent (essential oil from *Mentha spicata*) extended the shelf life by 12 days at 4 °C [111]. Similarly, a coating combined with banana starch and chitosan as polymeric compounds, sorbitol as a plasticiser, and *Aloe vera* gel as a biocontrol agent extended the shelf life by up to 15 days of storage at 8 °C and 70% RH [130]. Both studies significantly delayed fungal growth, the main microbial group responsible for strawberry spoilage.

Inline, chitosan coating with glycerol as a plasticiser and acetic acid as an additive extended the shelf life of strawberries by 15 days at 4 °C and 85% RH compared to the uncoated control. Acetic acid contributes to the formation of crosslinking between polymer chains, which increases the film density and its efficiency as a physical barrier. The coating reduced the physiological and biochemical changes during storage [131].

Still, edible food coatings can also be formulated without plasticisers. This contrasts with WPI, where plasticisers are essential to prevent the formation of brittle films and coatings; they are inherently brittle due to strong protein–protein interactions, necessitating the incorporation of plasticisers to enhance flexibility and avoid brittleness [132]. Cassava starch-based edible coatings (3%) extended the shelf life of strawberries by 3 days, from 9 to 12 days, at 5 °C. While effectively reducing weight loss, they did not preserve colour, contrasting the results observed with WPI films in this study. Furthermore, adding potassium sorbate (0.05%) to the cassava starch coating did not enhance its ability to control microbial growth [133]. In contrast, sodium alginate coatings (1%, *w*/*v*) in aqueous solution did not extend the shelf life of strawberries compared to the uncoated control, with both showing microbial damage from fungal growth after 7 days of storage at 4 °C and 95% RH. However, when the polymer was combined with an enhancer—nanocapsules loaded with *Lippia graveolens* essential oil—the multisystem coating demonstrated the ability to extend the shelf life by an additional 8 days [134].

Still focusing on coating solutions without plasticisers but incorporating multiple polymeric components, a multi-component edible coating made of bacterial cellulose, chitosan, and gellan gum extended the shelf life of strawberries by 6 days compared to uncoated strawberries stored at 20 °C and 80% RH. Especially for coatings with higher concentrations of chitosan, they controlled the growth of total mesophilic aerobic bacteria, moulds, and yeasts due to the antimicrobial properties of chitosan [135].

Thus, as reported in the literature, polymeric formulations without plasticisers either had no impact or provided a shelf life extension comparable to that observed in this study for coated strawberries. Additionally, the enhanced effectiveness in extending shelf life by adding enhancers and multipolymer combinations has been reported for both plasticised and non-plasticised polymeric formulations. This reinforces the synergistic interactions between polymers that have been described in the literature [119], suggesting that this effect is independent of the presence of a plasticiser.

## 4. Future Perspectives and Study Limitations

The results presented here, along with comparisons to other studies under similar conditions, underscore the potential of WPI in extending the shelf life of berries. Despite these promising outcomes, animal proteins remain underutilised in this area [126]. Specifically, WPI, a byproduct of the dairy industry, offers the added advantage of waste reduction, making it a sustainable alternative for strawberry preservation. Therefore, the preliminary findings here serve as a foundation for further enhancing WPI-based coatings to improve their effectiveness.

However, despite the promising results, the limitations of WPI-based coatings are well defined, particularly regarding their barrier properties [58], moisture sensitivity, and brittleness [136]. In this scenario, based on emerging articles for the chitosan polymer, whose studies are more abundant and are at a more advanced stage than that with WPI, increased crosslinking between whey protein chains could increase the density of the coating and, consequently, improve its barrier properties against water vapour and gas exchange, similar to the observations made with chitosan-based coatings [131]. Furthermore, thermal stability may be enhanced through this process, which would contribute to expanding the performance and stability of the films across a range of applications. Crosslinking could be tested using acids as well as transglutaminases. Furthermore, it is well-documented that WPI does not exhibit antimicrobial activity [137]. Thus, coatings that combine WPI and antimicrobial bioactive compounds may contribute to extending the shelf life of berries. Finally, it would be interesting to investigate the effect of coatings made with encapsulated WPI on the shelf life of strawberries, given the promising results obtained with polymeric nanoencapsulation [127]. Finally, sensory analysis of the WPI coating developed here, as well as of those improved versions based on it, will be crucial for assessing the practical applicability of these coatings in extending the shelf life of strawberries.

Still considering the limitations of WPI-coating use, it is important to highlight the restriction on the consumption of such coatings by individuals allergic to milk proteins, since whey proteins—especially α-lactalbumin (α-LA) and β-lactoglobulin (β-LG)—are the main allergens present in milk [138]. Regarding lactose content, WPI contains about 0.5–1.0% (*w*/*v*) lactose and has been suggested as a special-purpose food supplement suitable for consumption by most lactose-intolerant individuals [139,140]. This is because WPI contains 80–90% protein and can be pure enough to be virtually lactose-free [141]. However, individuals with a higher sensitivity to lactose should consider using plant protein-based coatings rather than those formulated with WPI.

Although this work is a preliminary study, it provides valuable insights into the functional behavior of WPI coatings and their impact on the commercial quality of fresh strawberries, offering practical evidence of their effectiveness under real storage conditions. The results contribute to a better understanding of WPI’s intrinsic potential for preserving high-moisture fruits and highlight relevant directions for future research, including morphological and microstructural analyses to deepen the comprehension of the film–fruit interface and its influence on shelf-life. Additionally, further sensory research evaluating attributes such as appearance, colour, taste, aroma, texture, and overall acceptability of the coated strawberries will be helpful for future industrial applications. In addition, the limitations of the present study also include subjectivity in the ratings of visual quality analysis.

## 5. Conclusions

Increasing the incorporation of WPI into the films reduced their solubility and moisture content, indicating improved integrity in high-moisture foods such as strawberries. Maintaining film integrity is crucial to protecting strawberries throughout storage and transportation. The increased tensile stress and elastic modulus, resulting from the presence of whey proteins, enhance the film’s resistance to traction at the expense of reduced elongation at break, making it less flexible. These properties are beneficial for minimising mechanical damage caused by impact and friction during transportation and prolonged storage.

Furthermore, the reduced flexibility suggests a denser film structure, emphasising water loss prevention in strawberries. This characteristic can help delay the shrivelling and deterioration of the fruit. Indeed, the WPI-coating effectively mitigated the strawberries’ mass loss, thus indicating protection against shrivelling and maintaining firmness and overall quality ratings above the acceptable limit for sale. Consequently, the coating lengthens strawberries’ shelf life according to commercial preferences during cold storage. Additionally, the decomposition onset temperature of the WPI-based films formulated here allows for adequate applications in fresh packaging or moisture-sensitive food products, as well as milder thermal processes, such as pasteurization. Still, the film-forming solution was more viscous and resistant to deformation at 4 °C than at 25 °C, indicating homogeneous coating of the fruit surface at room temperature and its adhesion at storage temperature.

Applying WPI films and coatings in this scenario could be a sustainable alternative to enhance global supply chain efficiency. Consequently, this could reduce logistics costs and waste. Overall, the findings suggest that WPI could be a viable alternative for producing biodegradable films and edible coatings with promising applications in berries. Additionally, this study lays the groundwork for future advancements in WPI-based films and coatings, which remain underexplored in the field of berry preservation, much like other animal-derived proteins.

## Figures and Tables

**Table 1:** Whey protein isolate and glycerol ratios used in the film-forming solutions.

Formulations	Whey Protein Isolate Concentration (%)	Plasticiser (Glycerol) Concentration (%)
F1	50	50
F2	60	40
F3	70	30
F4	80	20
F5	90	10

**Table 2:** Visual quality scores and descriptors for strawberries.

	Scores and Description				
	1	2	3 ^c^	4	5
	Very Poor	Poor	Acceptable	Good	Excellent
Colour ^a^	Very dark purplish-red; significantly overripe or senescent	Overripe; very dark red	Fully red	Fully light red	Three-quarters to fully light red
Shrivelling ^a^	Extremely wilted and dry	Severe shrivelling	Shrivelling is evident, and fruit show evident signs of moisture loss	Minor signs of shrivelling	Commercial fruit appears very fresh and turgid
Decay ^b^	100%, characteristic sporulation, the fruit is either partially or entirely rotten	75%, moderate to heavy mycelium growth	50%, spots with decay and some mycelium growth	25%, slight brown discolouration of the tissues, probable decay	0%, no visible changes in the tissues
Firmness ^a^	Extremely soft and deteriorated	Soft and leaky	Minor signs of softness	Firm but less turgid	Very firm and turgid

^c^ A score of 3 was considered the minimum acceptable quality before strawberries became unmarketable [25]. Modified from Kelly et al. [24]. ^a^ Nunes [26]. ^b^ Nunes and Delgado [43].

**Table 3:** Thermal stability of the films according to thermogravimetric analysis.

Formulation	WPI (%)	Gly (%)	T_d_ (°C)	T_max_ (°C)	%∆m
F1	50	50	274 ± 0.01 ^a^	327 ± 1.53 ^a^	80.70 ± 2.31 ^a^
F2	60	40	276 ± 0.58 ^a,b^	357 ± 14.0 ^a,b^	75.23 ± 0.48 ^a,b^
F3	70	30	277 ± 0.01 ^b^	371 ± 2.65 ^b^	57.29 ± 0.94 ^b^

WPI: whey protein isolate; Gly: glycerol; T_d_: temperature of initial decomposition; T_max_: temperature of maximum degradation rate; %∆m: mass loss percentage in decomposition range. Analyses were performed in triplicate, and the data are reported as the means ± SD. ^a,b^ Means within the same column with different superscripts indicate significant differences among formulations (Kruskal–Wallis test and Dunn’s post hoc test, *p* < 0.05).

**Table 4:** Effect of two temperatures (4 °C and 25 °C) on the power-law parameters of the WPI solution.

Temperature (°C)	*K *(Pa.s^n^)	n	*R* *^2^*
4	4.19 ± 0.0001 ^a^	1.062 ± 0.001 ^a^	0.999 ± 0.015 ^a^
25	2.25 ± 0.0001 ^b^	1.064 ± 0.001 ^a^	0.999 ± 0.018 ^a^

Values are presented as mean ± SD (standard deviation); Different letters indicate significant differences between temperatures (*p* < 0.05); The consistency index *K* is expressed in Pa.s^n^.

## Data Availability

The original contributions presented in the study are included in the article/Supplementary Material; further inquiries can be directed to the corresponding author.

## Supplementary Materials

The following supporting information can be downloaded at: https://www.mdpi.com/article/10.3390/foods14223980/s1, Figure S1: Representative picture of the macroscopic appearance of formulation F3 (70% whey protein isolate to 30% glycerol); Figure S2: Mass loss curves for whey protein isolate (WPI) films: (a) F1 (b) F2 and (c) F3. F1: formulation 1 (50% WPI and 50% glycerol—Gly); F2: formulation 2 (60% WPI and 40% Gly); F3: formulation 3 (70% WPI and 30% Gly); Figure S3: The fit by Power Law rheological model for the formulation F3 (70% whey protein isolate to 30% glycerol) at temperatures of 4 and 25 °C; Table S1: Mass loss percentage (%) of coated and uncoated (control) strawberries during storage for 8 days at 4 °C.
