# Supplementary material for: Biodegradable Films and Edible Coatings Based on Whey Protein Isolate for Extending the Shelf Life of Commercial Strawberries
Source: Foods. 2025 Nov 20;14(22):3980. doi: 10.3390/foods14223980 (PMC12652241; doi:10.3390/foods14223980)
Supplement: Supplementary file 1 [file foods-14-03980-s001.zip › foods-3573071-supplementary.pdf]

8

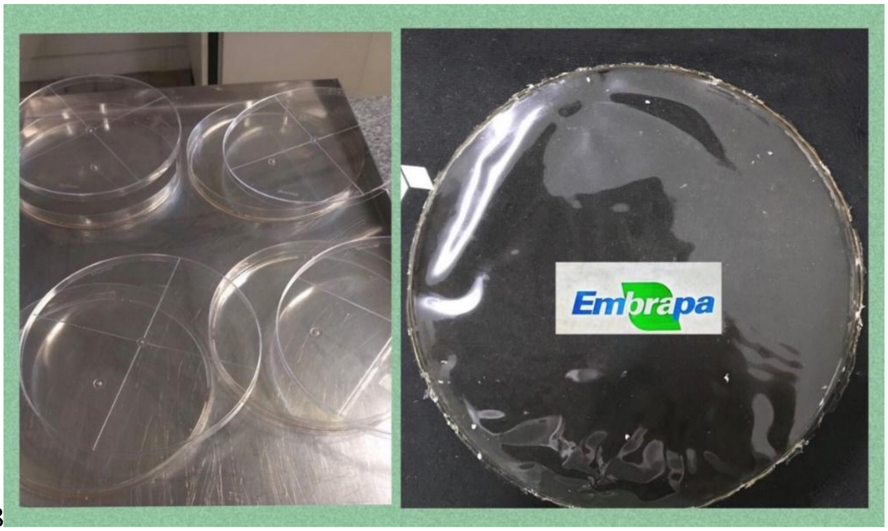

Figure S1. Representative picture of macroscopic appearance of formulation F3 (70% whey protein isolate to 30% glycerol). Empresa Brasileira de Pesquisa Agropecuária (Embrapa Agroindústria de Alimentos).

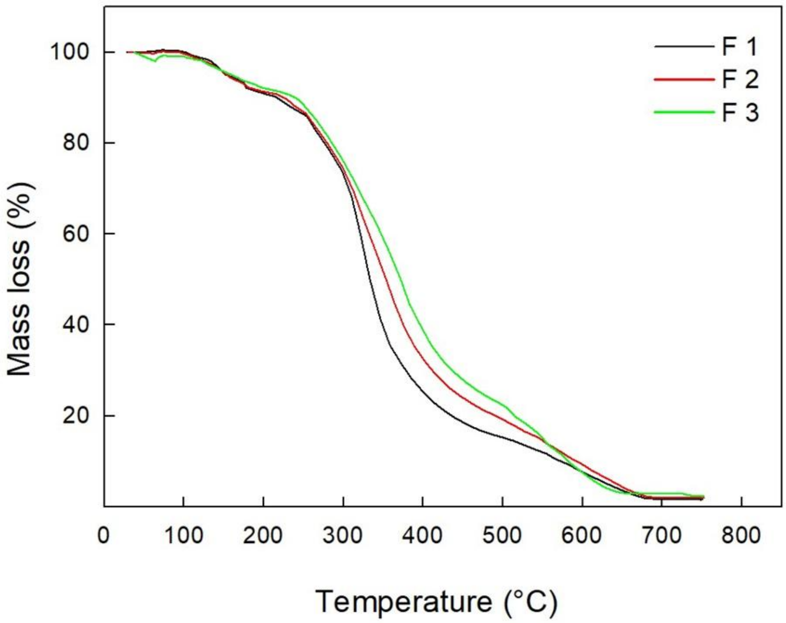

Figure S2. Mass loss curves for whey protein isolate (WPI) films: (a) F1, (b) F2, and (c) F3. F1: formulation 1 (50% WPI and 50% glycerol - Gly); F2: formulation 2 (60% WPI and 40% Gly); F3: formulation 3 (70% WPI and 30% Gly).

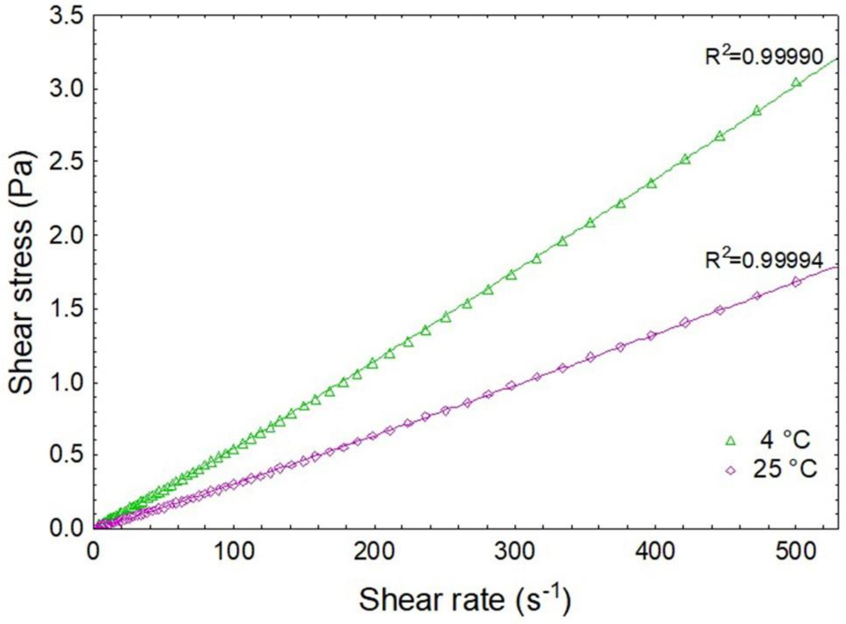

Figure S3. The fit of the Power Law rheological model for formulation F3 (70% whey protein isolate and 30% glycerol) at temperatures of 4 °C and 25°C.

Table S1. Mass loss percentage (%) of coated and uncoated (control) strawberries during storage for 8 days at 4 °C.

Samples	Storage time (days)				
	0	2	4	6	8
Control	0	$5.83 \pm 0.03^{a,A}$	$9.20 \pm 0.72^{b,A}$	$12.57 \pm 1.46^{c,A}$	$16.71 \pm 1.46^{d,A}$
Coated fruits	0	$2.56 \pm 0.15^{a,B}$	$6.15 \pm 0.54^{b,B}$	$9.75 \pm 0.93^{c,B}$	$13.22 \pm 1.22^{d,B}$

Analyses were performed in triplicate and the data are reported as the means ± SD.

^{a,b,c,d} Means within the same row with different superscripts indicate significant differences among storage days (Two-way ANOVA and Tukey's post-hoc test, $P < 0.05$).

^{A,B} Means within the same column with different superscripts indicate significant differences among treatments (Two-way ANOVA and Tukey's post-hoc test, $P < 0.05$).
